# Revolutionary Advances of Robotic Surgery in Urology Field

**DOI:** 10.14789/jmj.JMJ24-0008-R

**Published:** 2024-05-10

**Authors:** HISAMITSU IDE

**Affiliations:** 1 Department of Urology, Digital Therapeutics, Juntendo University, Graduate School of Medicine, Tokyo, Japan; 1 Department of Urology, Digital Therapeutics, Juntendo University, Graduate School of Medicine, Tokyo, Japan

**Keywords:** robotic surgery, da Vinci, hinotori, urology

## Abstract

The advent of robotic surgery has significantly impacted various surgical fields, particularly urology, gynecology, general surgery, and cardiac surgery. While the da Vinci robotic platform has been predominant over the past two decades, recent years have witnessed the emergence of new robotic platforms in Japan, now actively used in clinical practice. Currently, the available systems in Japan, alongside the da Vinci, include the Hinotori, Senhance, Hugo Ras, and Saroa surgical systems. This review focuses on comparing the notable functions of each system in urologic surgery, emphasizing the areas in which they differ from the da Vinci robotic platform. The development of new robotic systems is ongoing, promising not only cost reductions but also the introduction of innovative devices and educational systems. Soft robotics, which constructs robotic devices using soft, adaptable materials, has the potential to become central to the next generation of robotic surgery. Moreover, the collaboration between Artificial Intelligence (AI) and robotic surgery significantly contributes to increasing efficiency, accuracy, and safety in the medical field, with more innovative applications expected in the future.

## Introduction

In laparoscopic surgery, trocars with diameters ranging from 5 to 12 mm are inserted into the abdominal or retroperitoneal cavity. Carbon dioxide is insufflated to expand the abdominal cavity, allowing the necessary instruments to be inserted through the trocar ports for the operation. Compared to open surgery, laparoscopic surgery typically results in smaller surgical wounds, reduced blood loss, and shorter hospital stays. It can be performed either directly by the surgeon or with robotic assistance. However, surgeon-assisted laparoscopy has limitations such as restricted hand movements and limited maneuverability. The introduction of robotic systems marked a significant turning point in the field of surgery. Although conventional laparoscopic surgery, emerging since the 1980s, demonstrated the benefits of a minimally invasive approach, it had major drawbacks: limited movement confined to wrist rotation, minimal rotation at the forceps tip, and the absence of joint-moving instruments. These constraints made tasks like intracorporeal suturing extremely challenging. Consequently, training in laparoscopic surgery involved a steep learning curve, prolonging the training period for future surgeons and hindering the widespread adoption of laparoscopic techniques in the general medical community. In addition, the issues associated with scopists (doctors who operate the endoscope) in laparoscopic surgery can significantly affect the success of the surgery. When communication between the surgeon and the scopist is insufficient, it can lead to inadequate visualization during surgery. Close communication between the two is necessary to provide the precise field of view that the surgeon requires. There is also individual variation in the skill level of scopists, and a lack of experience or training can decrease the efficiency and safety of the surgery. Especially in complex surgeries, the skill of the scopist can directly impact the outcome of the surgery. During long surgeries, scopists can accumulate fatigue, which can lead to a decrease in attention and impairment in judgment, potentially leading to mistakes in operation. To address these issues, it is important to provide continuous education and training to enhance the professionalism of scopists, improve communication skills between surgeons and scopists, and strengthen the teamwork of the entire surgical team.

Robot-assisted laparoscopic surgery enables surgeons to operate using a robotic arm and console. Its advantages include high-precision operability and the amplification of the surgeon's fine hand movements, allowing for precise surgical procedures. Additionally, the surgeon can conduct operations with a highly flexible robotic arm inserted into the body cavity while observing three-dimensional endoscopic images on a console situated away from the patient. This setup provides a realistic view of organs and tissues. Robotic surgery reduces surgical complications such as bleeding, preserves organ function, and ensures more reliable anastomoses^1)^. In Japan, most procedures that can be performed laparoscopically are now covered by insurance in the field of urology. Indeed, at our facility, we have conducted robotic surgery for prostate cancer, bladder cancer, and renal cell carcinoma, and have reported achieving favorable results^2-6)^.

**Figure 1:**
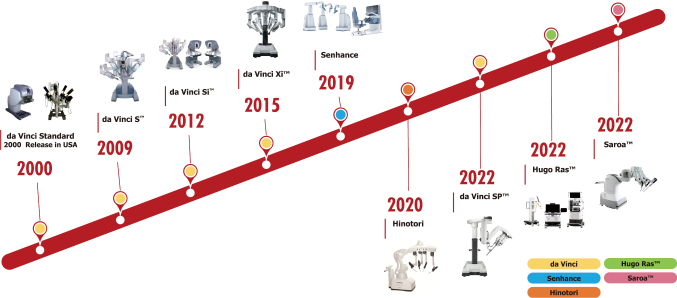
Time line of robotic surgical systems in Japan

While the da Vinci robotic platform has dominated the field of robotic surgery for the past two decades, recent years have seen the introduction of new robotic systems into clinical practice. Five new robotic systems have been commercialized and approved for clinical use in Japan, each designed to address the technical or cost limitations of the da Vinci platform. Currently available robotic systems in Japan include da Vinci, Hinotori, Senhance, Hugo Ras, and the Saroa Surgical System (Figure 1). Additionally, there is the ANSUR ‘collaborative assistant robot,' which specializes in assisting roles^7)^.

This review specifically focuses on the evolution and impact of these systems in the field of urology. With the development of Artificial Intelligence (AI) and further deepening of medical-engineering integration, robot-assisted devices including soft robotics have made significant progress, and their applicability in clinical practice is expected to continue expanding in the future.

## New multiport robotic surgical systems

The integration of robotics in surgery, driven by their capability for remote control and precision in repetitive tasks, has evolved over the past 50 years. Although the concept originated over five decades ago, practical application in surgery only began in the late 1980s^8)^. The da Vinci system, an endoscopic surgical support robot developed by Intuitive Surgical Inc. in the U.S., comprises three robotic arms, a laparoscope, and an operating console. The console also includes a pedal unit for various energy uses, such as monopolar and bipolar surgery, sealing devices, and automatic anastomosis devices. Approved by the U.S. Food and Drug Administration (FDA) in July 2000, the da Vinci system has undergone several upgrades (Figure 1). Currently, with quarterly revenues of $1.56 billion, Intuitive Surgical holds approximately 80% of the global market share for surgical robots^9)^.

**Figure 2:**
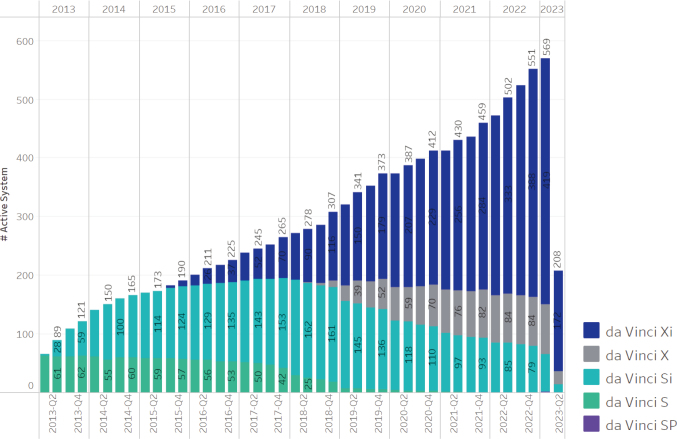
Number of da Vinci in Japan, provided by Intuitive Surgical Inc.

The da Vinci S model (2006) featured 3D high- definition camera vision, a simplified setup, and an interactive touch-screen display. Three years later, the Si model introduced dual console surgery and adopted firefly technology for real-time fluorescence imaging, enhancing tumor resection visualization^10)^. The Xi model boasts a redesigned patient cart for maximum mobility and flexibility, with a boom-mounted structure for docking from any angle and improved access around the patient. The arms have a wider internal range of motion, enhanced patient access, and reduced external collision interference. The Xi model also features compact flex joints for minimal arm interference and optimized arm positioning. Its 8mm endoscope provides lighter, brighter, and higher-resolution images. The da Vinci SP, a single-port surgical platform, facilitates varied surgical access with minimal arm interference and offers cosmetic benefits with smaller surgical wounds. The introduction of various da Vinci models in Japan has progressed rapidly since 2013. By 2023, 569 robots have been installed（Figure 2）. However, the high purchase and maintenance costs of the da Vinci system remain a barrier for many hospitals^11)^.

**Figure 3:**
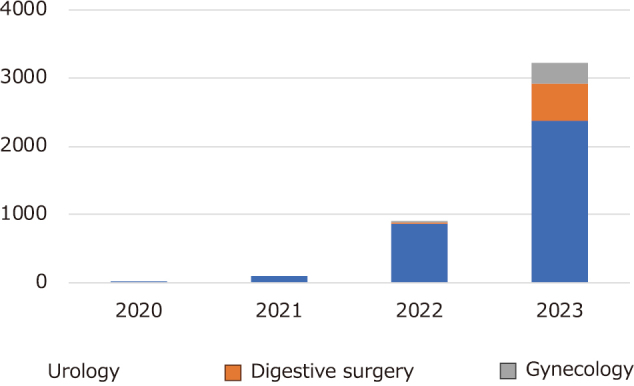
Hinotori cumulative number of cases, provided by Medicaroid

Japan's first robot-assisted surgery device, the “Hinotori Surgical Robot System,” developed by Medicaroid Corporation, was approved for manufacture and sale by the Ministry of Health, Labor and Welfare in August 2020, with insurance coverage following in September of the same year. The Hinotori system features a compactly designed surgical arm with eight axes, surpassing the seven axes of the preceding da Vinci system. This design minimizes arm-to-arm and arm-to-assistant interference, enabling smoother surgical operations. Robotic-assisted laparoscopic total prostatectomies using the Hinotori system have shown promising results^12)^. Currently, Hinotori is also utilized in surgeries within the surgical and gynecological fields^13-15)^. The author has performed robot-assisted laparoscopic radical prostatectomies using Hinotori. During the transition from the da Vinci to the Hinotori system, initial discomfort was noted in the docking system and forceps operation. However, these issues were resolved with each successive case. Additionally, research is underway in Japan to develop a teleoperative environment leveraging high-speed communication, AI, and other technologies^16-18)^. The Hinotori is considered an essential system in modern medicine, significantly improving the quality of surgeries with its precise operability, 3D visualization capabilities, reduction of surgeon fatigue, flexible approach, contributions to education and training, enhancement of patient comfort, potential for remote surgery, and possibilities for continuous technological innovation. These ongoing advancements are improving surgical outcomes. In the future, the integration with AI and the introduction of new sensing technologies, in addition to remote surgery, may enable even more advanced surgical assistance. Since 2020, the number of surgeries performed with the Hinotori has increased in urology as well as in gynecology and gastroenterological surgery. By 2023, it has been introduced in 44 facilities in Japan, conducting over 3,200 surgical cases (Figure 3).

The Senhance robotic system (Asensus Surgical, Durham, NC, USA), first introduced in 2012 as TELELAP Alf-X, received European approval in 2014^19)^. In Japan, Senhance gained approval from pharmaceutical affairs bodies and was included in insurance coverage in 2019. There are nine reports documenting the use of Senhance in Japanese surgical fields. The system's forceps movement is computer-controlled, with a stabilization mechanism to minimize tip blurring. The forceps tip movement is adjustable, featuring a ‘tactile feedback system' that simulates direct hand control. Additionally, a 3D high-definition camera can be operated using only eye movements^20-22)^.

The Hugo Ras system developed by Medtronic and launched in Japan in December 2022, features independently positionable arms and an open console, facilitating communication with operating room staff. This system allows multiple observers to simultaneously view the surgeon's operating screen. As of now, there is no report of Hugo being used in urology in Japan, with only one report in obstetrics and gynecology^23)^.

Ligasure is a device developed by Covidien (which has now been acquired by Medtronic) for sealing and cutting tissues. It is widely used in conventional laparoscopic surgery because it utilizes high-frequency energy to seal tissues and cut while minimizing bleeding. Its excellent sealing capability effectively reduces bleeding during surgery and shortens recovery time post-operation. When the Hugo RAS system is now developing in combination with Ligasure, it is expected to further enhance the precision and efficiency of robotic-assisted surgeries. The integration of Ligasure could become a notably distinct feature in surgeries using the Hugo RAS, potentially offering advantages over other robotic surgery systems. Specifically, the use of Ligasure may allow for more effective control of bleeding, improve the safety of the surgery, and reduce the duration of the procedure.

The Saroa Surgical System, equipped with a ‘tactile' sensation, received manufacturing and marketing approval in May 2023. Developed by Riverfield, a venture originating from Tokyo Institute of Technology and Tokyo Medical and Dental University, Saroa stands out for its pneumatically movable arms and pressure sensors that convey force directly to the doctor's fingertips. Conventional surgical support robots rely solely on visual information obtained from camera images, but Saroa has the ability to estimate the forceps' grasping force from control information and feed it back to the controller operated by the doctor. This allows the physician operating the robot to perform the procedure as if he or she were operating directly with his or her own hands, and is expected to realize safer and more precise surgery^24, 25)^. The Saroa system's patient cart and surgeon's console are about half the weight of traditional systems, offering ease of movement within facilities. The surgeon's console, similar to the Hugo RAS system, is an open platform. Although robotic surgeries using Saroa have commenced in the field of urology, no literature reports are available yet^26)^.

To enhance the safety of robotic surgery for each model, the Japanese Society of Endourology and Robotics and the Japan Society for Endoscopic Surgery etc. have established training systems, certification methods, and proctoring systems for performing robotic surgery on more than one model. These systems refer to education and training specialized for specific robotic surgery systems (for example, the da Vinci surgical system or other competing robotic systems). These training programs are designed to enable surgeons to learn how to use a specific robotic surgery system and to maximize their surgical skills within that system.

## Comparison of various robotic systems in urological surgery

In the field of urology, robotic-assisted laparoscopic surgery, characterized by magnified high- resolution 3D imaging, a wide range of motion in forceps joints, and significant operational freedom, is applicable for a variety of conditions. These include adrenal tumors, renal cell carcinoma, renal pelvis and ureter cancer, prostate cancer, bladder cancer, congenital hydronephrosis, and pelvic organ prolapse. When compared to open surgery and traditional laparoscopy, robotic-assisted laparoscopic surgery offers several advantages, such as reduced blood loss, improved perioperative outcomes, better functional preservation, shorter hospital stays, and enhanced quality of life. However, it also presents certain disadvantages:

• High operational costs, including maintenance and management expenses.

• In Japan, insurance reimbursement rates are low (no additional points for robotic usage).

• Significant non-reimbursable costs for materials.

• Lack of compatibility between the da Vinci system and other models in terms of cameras and surgical equipment.

• Complex requirements for surgical accreditation as stipulated by professional societies.

Although each new surgical robotic system is designed to address the technical or cost limitations of the da Vinci platform, a comparison of these surgical robots was conducted from the perspectives of setup, ease of use, device options, medical costs, surgical education, and teleoperation capabilities (Table 1). In terms of forceps variety, the da Vinci system is superior with thirty-nine types and also supports the use of sealing devices and automated anastomosis devices. Haptic feedback, a feature that simulates the sense of touch, is available with both the Senhance and Saroa systems. The console types of Senhance, Hugo Ras, and Saroa are open, which facilitates easier communication with the surgical assistant. A study comparing the Hugo Ras and da Vinci systems in robot-assisted laparoscopic radical prostatectomies reported no significant differences in total operative time or console time. However, it was noted that docking took longer with the Hugo Ras, which was attributed to its initial introduction phase^27)^. In robot-assisted laparoscopic partial nephrectomy (RAPN), Miyake et al. reported their surgical experience with 30 cases using the Hinotori system, achieving good results despite a short follow-up period. Additionally, a study comparing perioperative outcomes of RAPN using Hinotori and da Vinci was conducted. This analysis used propensity score matching and included 303 patients who underwent RAPN with da Vinci and 40 patients who underwent the procedure with Hinotori. The study found no significant differences in primary perioperative outcomes between the two systems, including operative time, robotic system use time, and warm ischemia time. The achievement of ‘trifecta' has been proposed as a comprehensive surgical outcome measure for partial nephrectomy. It evaluates whether the surgery meets all of the following goals: effective cancer control, preservation of renal function, and avoidance of complications^28)^. There were no significant differences in the rates of achieving trifecta, transection positivity, ischemia, and complication outcomes between the two groups. Additionally, changes in estimated glomerular filtration rate at 1 and 28 days post- RAPN were similar for both groups^29)^. This surgical robot Hinotori has several features that differentiate it from existing systems, including: software calibration of the trocar position without the need to attach the trocar, a compact operation arm with 8 axes of motion, and a flexibly positioned 3D viewer in the surgeon's cockpit. These advantages of hinotori are expected to reduce interference between the arms and between the arms and the doctor in a clean field, allowing the surgery to proceed smoothly. Importantly, the surgeon's cockpit reduces the burden on the surgeon with its ergonomic design. In Japan, various robotic systems are available for procedures such as robotic-assisted laparoscopic nephrectomy^30)^, robotic- assisted laparoscopic total bladder cystectomy, and robotic-assisted laparoscopic adrenalectomy^31)^. Further evaluation of each system's efficacy in these specific techniques is anticipated.

## Education of robotic surgery

Currently, most localized malignancies, with the exception of advanced cancers, can be treated using laparoscopic surgery. Surgeons, including urologists, are required to acquire safe and well- established techniques for robot-assisted laparoscopic surgery. The learning curve for robotic surgery techniques has been reported to be comparable in duration to that of traditional laparoscopic surgery, where forceps are manually handled, especially in the context of robotic-assisted laparoscopic total prostatectomy^32)^. In the past, the initial step in learning laparoscopic surgery involved studying surgical videos and practicing in a dry box. In the realm of robotic surgical education, in addition to video learning, training can also be conducted using a simulation image system on the console (Table 1). Various robotic platforms applied in surgery include the Revo-i Robotic Surgical System (Meere Company Inc., Seongnam, Republic of Korea), Avatera (avateramedical GmbH, Jena, Germany), and the Versius Surgical System (CMR Surgical, Cambridge, UK). As these technologies evolve, the global robotic surgical equipment market is expected to become increasingly competitive in the future^11)^. A crucial question now is what educational steps and systems will be implemented to train young surgeons who have limited experience in open and laparoscopic surgery.

**Table 1:** Comparison of robotic surgical systems in Japan

	Da Vinci (Xi)	Hinotori	Senhance	Hugo Ras	Saroa
Types of forceps	39	11	42	8	6
Haptic feedback	╳	╳	〇	╳	〇
Simulator	〇	〇	〇	〇	╳
Teleoperation capability	╳	Under development	╳	╳	Under development
Consol type	Close	Close	Open	Open	Open
Cost of unit (List Price: million yen)	275	235	200	230	Open price

As of January 4, 2024

## Future direction of robotic surgery

The integration of AI and robotic surgery represents a significant advancement in medical technology of urology field^33)^. Here are the key aspects of how AI and robotic surgery are working together. AI utilizes image analysis technologies to extract detailed information from medical images such as MRI, CT scans, and X-rays. This enhances the accuracy of diagnostics in the preoperative planning phase, thereby enhancing the precision of surgeries. Additionally, AI algorithms can analyze patient-specific data to determine the most suitable surgical approach, enabling the creation of customized surgical plans for each patient and minimizing risks. Robotic surgical systems use data provided by AI to perform more accurate and precise movements during surgery, allowing surgeons to operate with high precision in tight areas or on minute tissues. During surgery, AI can analyze data in real-time, providing critical information to surgeons and enabling more informed decision- making, thus improving the safety and efficacy of the surgery. Furthermore, AI systems can learn from past surgical data to provide insights for improving the success rates of future surgeries^34-36)^.

This promises advancements in surgical techniques and better patient outcomes. Even in the post-operative period, AI can monitor patient recovery and recommend necessary interventions, enhancing the overall quality of care. Future directions for robotic surgery training may include the use of technologies such as AI and machine learning for real-time feedback, remote mentoring, and augmented reality platforms, aiming to reduce costs and overcome geographic limitations^37)^.

In current robot-assisted surgical approaches, surgeons mainly operate rigid, metallic robotic devices, resulting in limited operability. Soft Robotics, characterized by the use of flexible materials like silicone, rubber, and plastic, allows operation in complex shapes and convoluted environments. This field aims to emulate biological flexibility and adaptability, achieving more natural and flexible movements compared to traditional robotic designs that use metal for both the robot's links and joints. This enables changes in shape and rigidity that were previously impossible. If control issues can be resolved, soft robotics could become central to the next generation of robotic surgery^38, 39)^. Its flexible structure offers greater safety in interactions with humans and the environment, making it suitable for medical applications and collaborative work. Soft robots are expected to adapt and deform in response to their environment, akin to biological muscles. In the medical field, these characteristics enable flexible surgical robots to operate in narrow spaces and perform precise tasks challenging for traditional rigid robots. Soft robotics is a nascent field with challenges like durability, precise control of force, programming complex movements, and improving energy efficiency^40)^.

**Figure 4:**
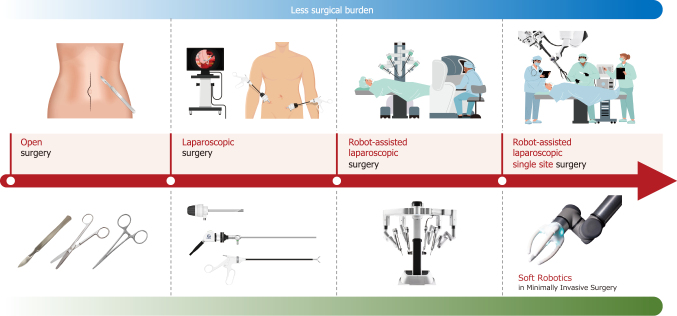
Evolution of robotics in minimally invasive surgery

In the future, soft robotics has the potential to transform the role of robots in many medical fields by achieving more natural and human-like movements and improving interactions with humans, especially when combined with advanced AI. This innovative field brings a new dimension to robot technology with its flexibility, safety, and adaptability, potentially enabling a future where robots are deeply integrated into human life. Despite the technical challenges, advancements in this medical field are expected to push the boundaries of robotics, promoting the development of more flexible and interactive robots (Figure 4).

## Conclusion

Twenty-four years have elapsed since the introduction of the da Vinci robotic surgery system in Japan. Several areas in the robotic surgery platform, including newer models, require improvements. These improvements include enhancing communication with assistants through open consoles, improving modularity, ensuring compatibility with other models, reducing equipment size, and lowering costs. Additionally, the insurance system in Japan presents barriers to robotic surgery, such as low reimbursement rates for robotic procedures, the absence of additional fees for robotic surgery depending on the procedure, high costs of non-reimbursable materials, and the complexity of surgical accreditation requirements set by academic societies. As robotic surgery continues to mature clinically and as technology evolves, we can anticipate the development of new systems that incorporate artificial intelligence technology in various surgical fields.

## Funding

No funding was received for this manuscript.

## Author contributions

HI planned this manuscript, collected the appropriate literature information, and drafted the manuscript. The author read and approved the final manuscript.

## Conflicts of interest statement

The author declares that there are no conflicts of interest.
